# Viral subversion of the cell polarity regulator Scribble

**DOI:** 10.1042/BST20221067

**Published:** 2023-01-06

**Authors:** Airah Javorsky, Patrick O. Humbert, Marc Kvansakul

**Affiliations:** 1 Department of Biochemistry and Chemistry, La Trobe Institute for Molecular Science, La Trobe University, Melbourne, Victoria 3086, Australia; 2 Research Centre for Molecular Cancer Prevention, La Trobe University, Melbourne, Victoria 3086, Australia; 3 Department of Biochemistry and Pharmacology, University of Melbourne, Melbourne, Victoria 3010, Australia; 4 Department of Clinical Pathology, University of Melbourne, Melbourne, Victoria 3010, Australia

**Keywords:** cell polarity, PDZ domain, Scribble, virology

## Abstract

Scribble is a scaffolding protein that regulates key events such as cell polarity, tumorigenesis and neuronal signalling. Scribble belongs to the LAP family which comprise of 16 Leucine Rich Repeats (LRR) at the N-terminus, two LAP Specific Domains (LAPSD) and four PSD-95/Discs-large/ZO-1 (PDZ) domains at the C-terminus. The four PDZ domains have been shown to be key for a range of protein–protein interactions and have been identified to be crucial mediators for the vast majority of Scribble interactions, particularly via PDZ Binding Motifs (PBMs) often found at the C-terminus of interacting proteins. Dysregulation of Scribble is associated with poor prognosis in viral infections due to subversion of multiple cell signalling pathways by viral effector proteins. Here, we review the molecular details of the interplay between Scribble and viral effector proteins that provide insight into the potential modes of regulation of Scribble mediated polarity signalling.

Cell polarity refers to the differential distribution of macromolecules within a cell that enables the proper orientation of the cell in specific directions. This ability is essential for correct tissue and organ development and function [1], and impacts key cellular processes ranging from proliferation regulation, neuronal signalling and signalling complex formation at particular subcellular sites. Cell polarity is controlled by the interplay of several large multi-protein complexes including the Scribble complex, and their assembly, interactions and cellular localisation control crucial polarity events. Unsurprisingly the dysregulation of these mechanisms and regulation of cell polarity underpin the development of a range of diseases, including viral infections [2]. The focus of this review is on the interplay of virus-encoded proteins with Scribble and components of the Scribble complex. The interplay of Scribble complex components with endogenous cellular interactors and the implications for non-viral diseases have been reviewed by others [3].

The impact of cell polarity on cell and tissue shape is illustrated for example by the axonal-dendritic direction of neurons, the migration and asymmetric division of mesenchymal cells and the apical-basal orientation or planar polarity of epithelial cells (Figure 1) [3]. Overall, these phenomena are underpinned by four main types of cell polarity: (1) asymmetric cell division (ACD), (2) planar cell polarity (PCP), (3) apical-basal cell polarity (ABCP) and (4) front-rear cell polarity (FRCP) (Figure 1) [3].

**Figure 1.**
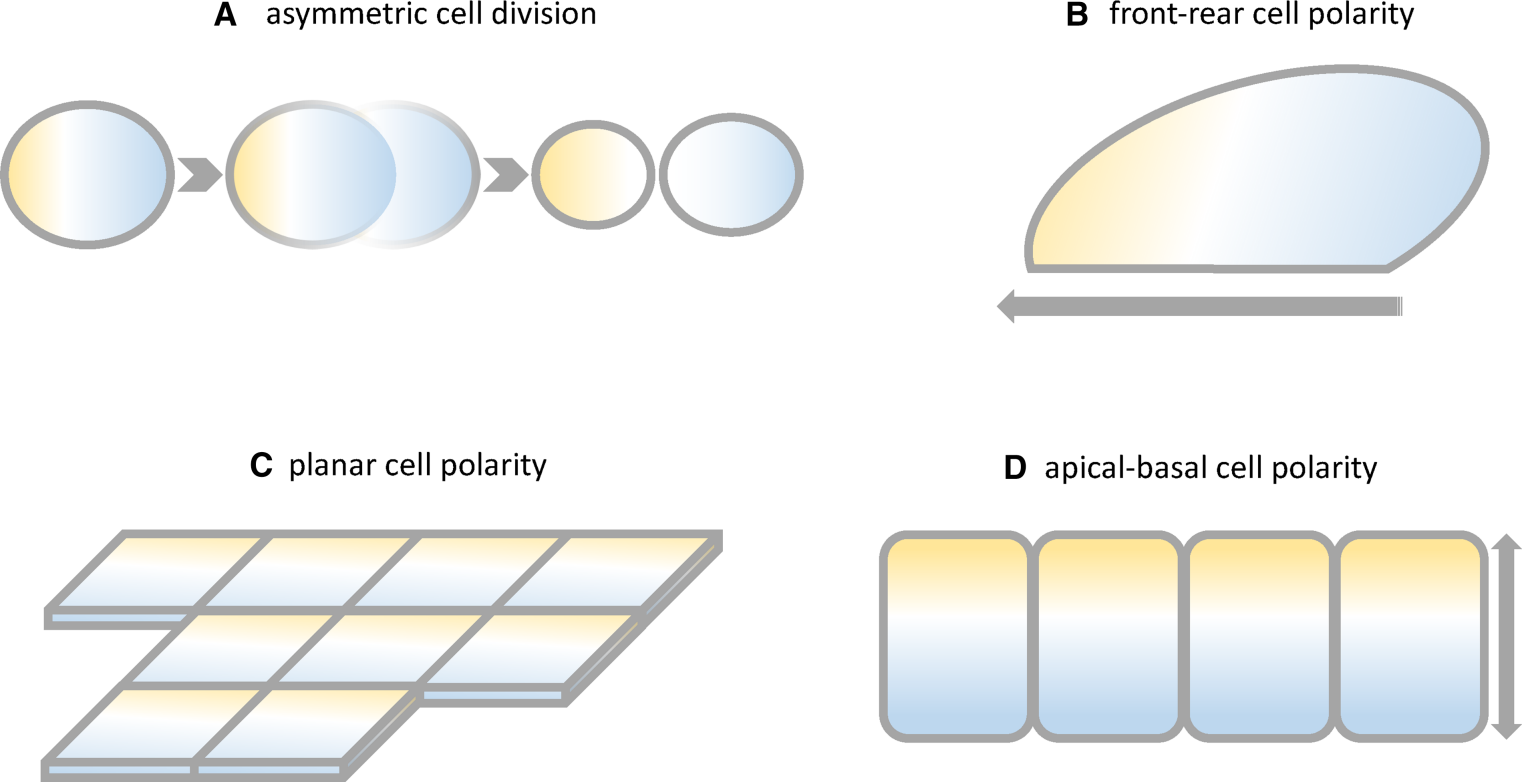
Schematic diagram of the four main types of cell polarity. Yellow and blue are putative polarity proteins shown in the polarised localisation required for them to control cellular orientation. (**A**) Asymmetric cell division of a stem cell. (**B**) Front-rear cell polarity of a migrating fibroblast. (**C**) Apical-basal cell polarity of epithelial cells (**D**) Planar cell polarity of epithelial cells in a top view.

For apical-basal as well as migrating and asymmetrically dividing polarity cell types, three key protein complexes act in concert that exists in an antagonistic relationship; the PAR complex, the Crumbs complex and the Scribble module (Figure 2) [3]. The Scribble module is generally localised below tight junctions near the basolateral cell membrane (Figure 2) and has been shown to interact with several viral proteins, which disturb the formation of adherens junctions [4]. The Scribble module is a target of tumorigenic viruses through the deployment of virulence and other viral effector proteins including those that are oncogenic (Figure 2) [5].

**Figure 2.**
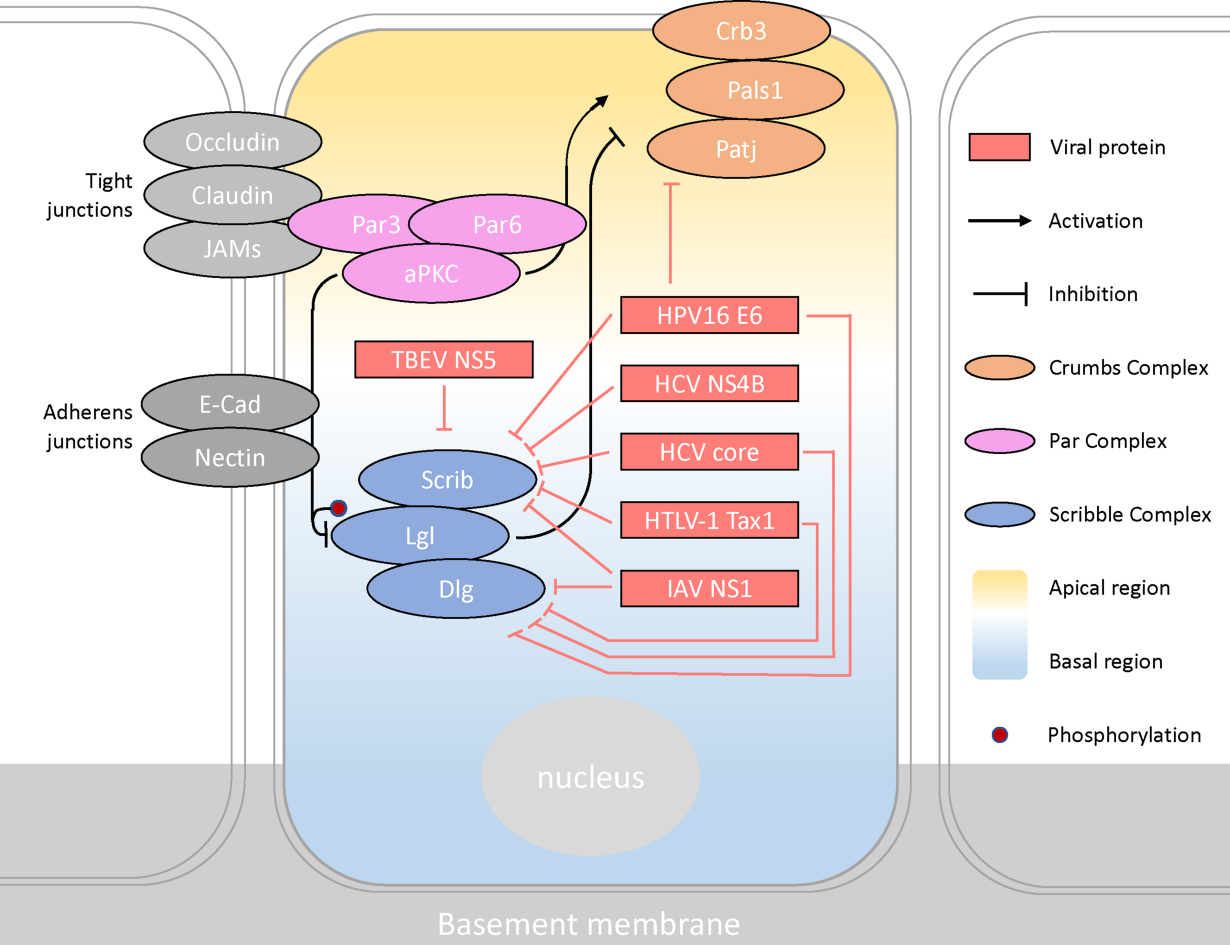
Schematic diagram of the viral proteins interacting with protein complexes involved in regulating cell polarity. In apical-basal cell polarity, cellular regions are divided into the apical, basal and lateral regions. Viral effector proteins (red) interact with polarity proteins. The Crumbs complex (orange) is composed of Crb, Pals and Patj and the Par complex (pink) composed of Par3, Par6 and aPKC are in the apical region. The Scribble module (blue) includes Scribble, Dlg and Llg in the lateral-basal region. Mutual antagonistic interactions exist between the complexes to establish the orientation of the cell. Figure adapted from Humbert et al. (2003).

Originally identified in *D. melanogaster*, Scribble mutant embryos displayed a corrugated morphology riddled with holes in the epidermal organisation, hence the name *scribble* [6]. The loss of Scribble triggers changes in the localisation of apical proteins, leading to disturbance of basolateral adherens junctions and resulting in the disruption of cell polarity in epithelia, neurons, and T-cells [7–9]. Consequently, hyperproliferation occurs, thus implicating Scribble as a determinant of cell growth and tumour suppression [3,10]. Other roles of Scribble include cell migration and adhesion, membrane and cytoplasmic signalling, GTPase and Kinase activation, neuronal development and apoptotic process involved in morphogenesis [7,11–16].

The human Scribble module comprises Scribble (SCRIB) and one of four Dlg homologues (DLG1–4) and two Lgl homologues (LLGL1 and 2), which are all large adaptor proteins featuring several protein interaction domains that allow them to bind to e.g. kinases and phosphatases as a means of regulating cell signalling. However, their functions are also dependent on their cellular localisation [3,17]. Scribble belongs to the LAP (leucine-rich repeats and PDZ domain) protein family, with sixteen LLRs (Leucine-rich repeats), two LAP-specific domains (LAPSDa and LAPSDb) and the four PDZ domains (PDZ1, PDZ2, PDZ3 and PDZ4) (Table 1) [18]. The N-terminal LRR domain and four PDZ domains are key to a range of protein–protein interactions, often affecting the localisation of the Scribble complex within the cell (Table 1) [12]. In particular, the PDZ domains have been identified to be crucial mediators for the vast majority of Scribble and ligand interactions, particularly through the C-terminal PDZ Binding Motif (PBM) encoded in interactors (Table 1) [3,17,19–29]. PBMs are categorised into three distinct classes, with class 1 PBMs featuring a consensus sequence of X-T/S-X-ϕ_COOH_ (where X is any residue and ϕ is any hydrophobic residue), whereas the class 2 consensus sequence is X-ϕ-X-ϕ _COOH_ and class 3 is X-D/E-X-ϕ _COOH_ [30–32]. The majority of Scribble ligands such as β-pix, APC and vangl feature a class 1 PBM, an observation that also applies to viral interactors such as HPV E6, HTLV-1 tax, IAV NS1 and TBEV NS5. Only one known viral interactor, HCV NS4B, utilises a class 2 PBM [19–23,26–28].

**Table 1:** Interactors of Scribble

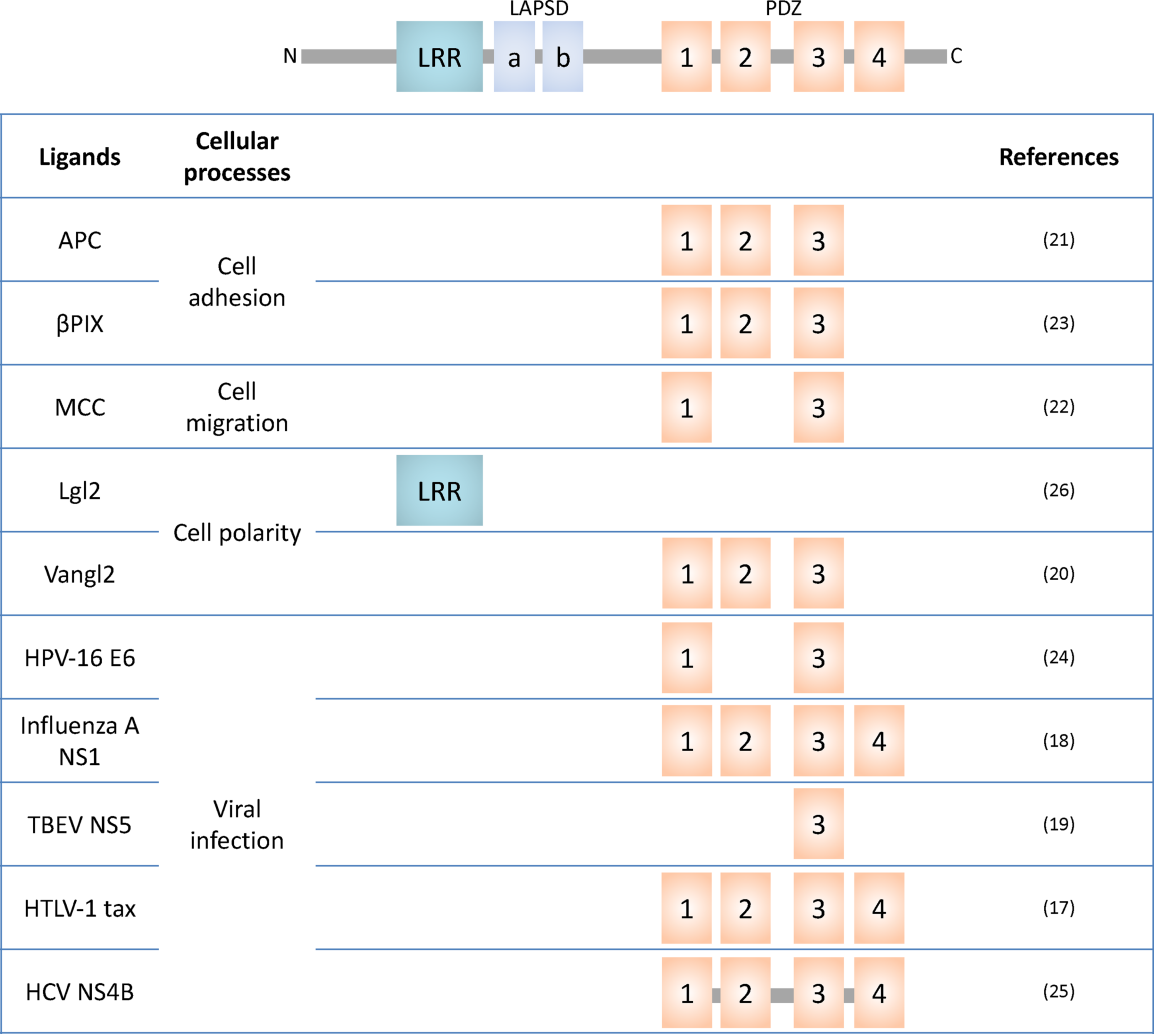

Interacting domains that bind to their respective ligands and their cellular functions are outlined. Starting from the N-terminus, Scribble contains an LRR domain (cyan), LAPSDa and b (light blue) and four PDZ domains (orange).

Numerous studies have shown that PBM/PDZ interactions are a driving factor for ligand binding flexibility and spectrum, but also impart specificity control [33] (Figure 3). Due to the wide range of cellular functions influenced by Scribble, several viral proteins have evolved to contain PBMs that interact with Scribble PDZ domains. These viral proteins include the Human Papillomavirus (HPV) E6, Human T-lymphotropic virus 1 (HTLV-1) Tax, Tick-borne encephalitis virus (TBEV) non-structural 5 (NS5), Hepatitis C virus (HCV) core protein and NS4B protein as well as Influenza A virus (IAV) NS1 (Table 1) [28,34–38].

## Influenza A virus (IAV) NS1 protein

Influenza A virus is part of the family of orthomyxoviruses, which feature a negative-strand RNA genome, and infects the respiratory epithelia of birds and mammals [39]. In recent studies, influenza A viruses have been found to encode PBMs in the NS1 (non-structural 1) protein that targets a range of PDZ domains to aid dissemination in the host and transmission to new hosts as a means of enhancing viral replication [4]. Therefore, of particular interest have been reports of the binding of Influenza A NS1 to Scribble [36,40].

**Figure 3.**
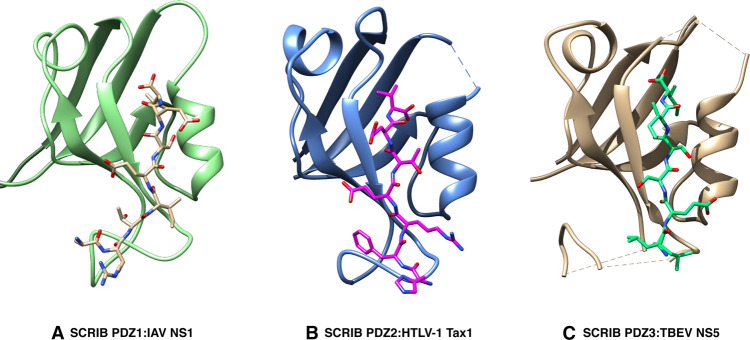
The crystal structures of SCRIB PDZ domains with viral ligand PBMs. (**A**) SCRIB PDZ1 (green) is shown as a cartoon, and influenza A NS1 ESEV peptide (gold) (pdb: 7QTO). (**B**) SCRIB PDZ2 (blue) shown as a cartoon HTLV-1 Tax1 PBM peptide (magenta) (pdb: 7QRT). (**C**) SCRIB PDZ3 (gold) is shown as a cartoon, and TBEV NS5 PBM peptide (green) (pdb: 7QSA). All three viral PBMs dock in between the second α-helix and the second β-strand, in which the last c-termini four amino-acids form a β-strand antiparallel to the second β-strand. Conformational flexibility arises beyond the last four C-terminal residues in the direction of the N-terminus, which is mediated by interactions of the PBMs with the loop region between the second and third β-strand.

The 26 kDa NS1 influenza A protein uses multiple mechanisms to aid in viral replication, such as preventing polyadenylation and splicing of cellular mRNA [41]. Four major PBM sequences located at the C-terminus have been isolated from human influenza A NS1: RSKV (seen in H3N2), ESKV (found in H9N2, H5N1, H5N2), KSEV (associated with H1N1) and ESEV (from H5N1) [36]. These sequences all contribute to the pathogenicity of their corresponding strains. However, animal studies that investigated survival rate and other determinants of pathogenicity revealed variations in virulence, potentially due to differences in PBM sequences of NS1 found in different strains [42,43]. Studies using pull-down assays have shown that ESEV PBM of H5N1 NS1 binds to the PDZ domains of SCRIB, DLG1 and several other PDZ domain-containing proteins (MAGI-1, MAGI-2 and MAGI-3) [40]. Initial *in vitro* data suggested that NS1 engaged both human Scribble PDZ1 and PDZ2 domains together [36]. However, isothermal titration calorimetry (ITC) studies together with crystal structures (Figure 3A) revealed that the ESEV PBM sequence has the ability to bind to all four PDZ domains of Scribble with PDZ4 showing the tightest affinity (*K*_D_ = 12.2 µM), whereas PDZ1 was the weakest interactor (*K*_D_ = 21.4 µM) [20]. It remains to be shown if NS1 binds all four Scribble domains *in vivo.* In infected cells, NS1 harbouring the ESEV PBM localised Scribble into cytoplasmic puncta concentrated in perinuclear regions, and also protected infected cells from apoptosis by directly targeting Scribble, which in turn also enhanced viral replication *in vitro* [36]. The disruption of Scribble, Dlg proteins and other PDZ-containing polarity proteins also disables tight junction (TJ) formation and benefits viral replication by enhancing virus dissemination to uninfected host cells, thus supporting viral spread [40]. NS1 has also been shown to inhibit antigen-presenting cell (APC) functions by interacting with Scribble and DLG1 [44]. The severity of the H5N1 influenza A virus infection can range from diarrhoea, encephalitis, cytokine storm, oedema within the lungs, organ failure and death [45].

## Tick-borne encephalitis virus (TBEV) NS5 protein

TBEV is a zoonotic virus and is often transmitted to humans via the bite of infected ticks leading to severe encephalitis, with an associated mortality rate of 20–30% [46,47]. A member of the family of Flaviviruses, TBEV features a single-stranded RNA genome that encodes a single polyprotein during the viral life span [46]. Post-translational modification and processing of the polyprotein yields three structural and seven non-structural (NS) proteins in the order C-prM-E-NS1-NS2A-NS2B-NS3-NS4A-NS4B-NS5 [48]. NS5 is the largest (800–900 amino acids) and most conserved of the *Flaviviridae* proteins [49]. NS5 harbours an N-terminal methyltransferase (MTase) and a C-terminal RNA-dependent RNA polymerase (RdRp) that are required for the capping and synthesis of the viral RNA genome, respectively [50].

NS5 was initially shown to feature two PBM sequences, with an internal PBM found within the MTase domain demonstrated to be important for binding to SCRIB via the PDZ4 domain [35,51]. Internal PBMs are uncommon, with the vast majority of PBM sequences identified at the C-termini of PDZ domain interacting proteins, however, internal PBM sequences are increasingly recognised to be more prevalent than previously believed, thus underscoring the plasticity of PDZ domains that can be exploited for molecular recognition and regulation of interactions [52–54]. The identification of the Scribble PDZ4 domains as the primary target of NS5 was challenged by recent studies that revealed Scribble PDZ3 as the main interactor with the canonical C-terminal PBM of NS5. Furthermore, no interactions of the internal PBM with any of the SCRIB-encoded PDZ domains were reported [21]. These findings were supported by a crystal structure of SCRIB PDZ3 bound to the canonical C-terminal PBM of NS5 (Figure 3B) [21]. The competing studies employed differing experimental methodologies and used PBM sequences of differing lengths, which may account for the discrepancy between the findings [21,51]. On a cellular level, the interaction with NS5 causes Scribble and DLG1 to relocate to the plasma membrane and disrupt synaptic membrane exocytosis-2 (RIM2), a PDZ containing neuronal-specific protein, and Zonula occludens-1 (ZO-1) involved in the interferon-mediated JAK/STAT signalling; presumably to evade the innate immune responses [35,55]. The interaction of NS5 and Scribble has also been shown to impair neurite outgrowth, possibly by disrupting the Scribble/Rac1/βPIX scaffold thereby hindering cytoskeleton changes [56]. This highlights the scope of Scribble's involvement in apicobasal cell polarity, with the capacity to affect signalling regulation and neuronal differentiation being exploited as an opportunity by viruses to support their replication.

## Hepatitis C virus (HCV) core (C) protein and NS4B protein

Hepatitis C viruses are single-stranded RNA viruses and belong to the family of *Flaviviridae*. They infect hepatocytes and are the causative agent of hepatitis C, as well as a known cause of steatohepatitis, fibrosis, cirrhosis, and hepatocellular carcinoma [57].

The positive-sense single-stranded RNA genome encodes a single precursor polyprotein, processed by cellular and viral proteases into 10 structural and non-structural proteins; core, E1, E2, p7, NS2, NS3, NS4A, NS4B, NS5A, and NS5B. The core protein is also known as structural capsid protein (C) and is a multifunctional protein that mainly functions to form the nucleocapsid, and together with envelope glycoproteins (E1 and E2) forms the structural components of the virion [58]. Furthermore, the HCV C protein acts by protecting the RNA while it passes from one cell to another by encapsulating HCV RNA in the cytoplasm, which then interacts with E1 and bud into the ER lumen, then released, presumably via the secretory pathway [59]. The monomeric, mature form of HCV core is a 21 kDa protein that possesses two distinct domains, D1 and D2, distinguished by different amino acid compositions and hydrophobicity profiles [60].

Currently, it is not clear whether the core protein has a distinct active PBM, however, it is able to inhibit the SHIP2 phosphatases, causing down-regulation of DLG1 and Scribble at the basolateral membrane [37]. Another important HCV protein that harbours a confirmed class I PBM and interacts with Scribble is the 27 kDa HCV NS4B protein, which features an N-terminal domain, a C-terminal domain, a central transmembrane domain and a class 1 PBM at the C-terminus [28,61]. This interaction was shown to induce the degradation of Scribble via the proteasome-ubiquitin pathway, which disrupts colony formation of transfected cells [28]. Furthermore, NS4B disrupts Scribble through an up-regulation of the epithelial to mesenchymal transition (EMT) master transcription factor SNAI1, which affects the Scribble-Hippo-PI3k/AKT pathway thus inducing EMT [62]. Co-IP assays demonstrated a direct interaction with a single protein chain featuring PDZ1, PDZ2 and PDZ3 or PDZ2, PDZ3 and PDZ4, but not with single individual Scribble PDZ domains [28]. The observed requirement of at least three contiguous PDZ domains of Scribble suggests inter-domain regions between neighbouring Scribble PDZ domains may play key roles in modulating the interaction with NS4B, underscoring the ability of viruses to deploy multiple molecular strategies to perturb Scribble-mediated signalling, and in the case of HCV in combination with hepatic inflammation contribute to cellular transformation.

## Human papilloma virus (HPV) E6 protein

HPV is recognised as the causative agent for cervical cancers as well as a contributor to anogenital and oropharyngeal cancers [63]. The ability of the virus to induce malignancies is dependent on two oncogenic proteins, E6 and E7, which induce and maintain cellular transformations of keratinocytes [64,65]. Although these proteins have been characterised to interact with a wide range of proteins and transcription factors to inhibit key signalling pathways including apoptosis, they can also target PDZ domain harbouring proteins to disable cell polarity and induce hyperplasia [66].

The ∼19 kDa (early expressed) E6 protein has multiple functions to abrogate pro-apoptotic responses including the ability to redirect ubiquitin ligases, which allows for proteasomal degradation of the crucial oncogene p53 [66,67]. Interestingly, low-risk HPV induced by HPV strains encoding for E6 lacking a PBM causes benign lesions, in contrast with high-risk strains including the most virulent HPV 16 and 18 that encode for a PBM at the E6 C-terminus [68]. High-risk E6 targets several PDZ domains-containing polarity proteins, such as DLG1, Scribble, PATJ and MAGI-1, for ubiquitin-mediated degradation via the proteasome [68–73]. It was shown that E6 dimerises to enable the degradation of p53 via direct interaction with the ubiquitin ligase E6AP [26,74], suggesting that a similar mechanism may apply to PDZ domain containing proteins that bind to the E6 PBM [75,76]. E6 has also been demonstrated to trigger a relocalisation of Scribble and DLG1 from the cellular membrane into the cytoplasm [77]. This allows E6 to further stabilise, maintain and increase in infected cells, particularly when binding to Scrib [78,79]. Interestingly, slight PBM variations can significantly alter ligand specificity [80], even a minor change of the last amino acid such as a change of the sequence ETQL (from HPV-16) to ETQV (from HPV-18) changes E6 binding preference from Scribble to DLG1 [38]. To date, ETQL has been shown via GST-pull downs to bind to individual PDZ1, 3 and 4 domains of Scribble but not to PDZ2 [81]. However, a recent study [27] demonstrated that ETQL and ETQV binds to individual PDZ1, 2 and 3 but not PDZ4 domains using ITC, The differences in experimental methodologies and constructs may underpin the discrepancies with reported finding. Strikingly, an increase in the number of PDZ domains targeted by E6 correlates to an increase in E6 cancer-causing potential, with HPV-16 and HPV-18 PBMs featuring the most promiscuity with respect to their PDZ domain target selection [82]. E6 interactions with Scribble and DLG1 was limited to PBMs of strong cancer association (types 16, 18, 31, 33, 35, 51) [5], suggesting that interference with Scribble module signalling supports transformation and enhances malignancy, as seen in cellular transformation assays [83,84].

## Human T lymphotrophic virus-1 (HTLV-1) Tax protein

HTLV-1 is a single-stranded RNA T-lymphotropic tumour virus and has been identified as a direct cause of adult T-cell leukaemia. The development of adult T-cell leukaemia is dependent on the expression of the oncogenic viral protein Tax for the maintenance of the malignancy [85]. Tax encodes for a C-terminal PBM that binds to numerous polarity proteins and leads to the aggregation or mislocalisation of DLG1, Scribble, MAGI-1 and MAGI-3 [86–89]. These interactions disturb host cell control over cell adhesion, proliferation, and signalling. Using humanised CD34+ mice it was shown that Tax PBM enhances HTLV-1-induced T-cell proliferation [86]. Immunofluorescence (IF) and in situ proximity ligation (PLA) assays both revealed that Tax and Scribble co-localise in large aggregates, near the cell membrane. In contrast, such aggregates were not detected when using a mutant of Tax that lacked the C-terminal PBM, confirming the ability of Tax PBM to sequester and redirect Scribble [86,90]. Others reported altered subcellular localisation of DLG1 and Scribble in 293T cells, as evidenced by a shift from a detergent soluble fraction into an insoluble fraction [91]. The precise mechanism how Tax interacts with cell polarity regulatory proteins was initially shown to involve an interaction of the Tax PBM with PDZ2 and PDZ4 of Scribble, but a second study demonstrated via the use of fluorescence polarisation assays that PDZ2 and PDZ3 were the primary interactors [34,92]. More recently it was shown that the Tax PBM binds all four individual PDZ domains using ITC, supported by crystal structures of complexes of SCRIB PDZ1:Tax1, SCRIB PDZ2:Tax1 and SCRIB PDZ3:Tax1 (Figure 3C) [19]. Notably, all conflicting studies used difference experimental approaches, protein constructs and expression tags, which may be responsible for differing results. The mislocalisation of SCRIB upon binding of Tax PBM is possibly the cause for the morphological changes that affect the actin cytoskeleton, and help the sustained cellular proliferation leading to tumorigenesis. Furthermore, the significance of the Tax1 PBM is highlighted by its absence in the Tax protein of the non-oncogenic HTLV-2 [34], underscoring the profound difference the presence of the Tax1 PBM has on HTLV-driven tumorigenesis.

## Conclusion

Considering the pivotal role that cell polarity plays in correct tissue architecture as well as cellular function, it is not surprising that polarity regulation is subverted by numerous viruses [2,93]. There is considerable complexity in the protein interaction networks underlying Scribble module-mediated regulation of signalling, and this impacts a diverse set of crucial pathways including apoptosis, cell proliferation as well as innate and acquired immunity [35,36,40,42,81]. Although recent studies have shed light on the potential molecular mechanisms underlying the subversion of Scribble signalling by viral effector proteins, particularly via PDZ–PBM interactions, no structures of full-length complexes have been determined to date [19–21], a gap that remains to be closed in the future. In light of the comparable affinities observed for viral and endogenous Scribble interactors, it seems unlikely that competition between different interactors alone is responsible for the substantial disruption of Scribble signalling. Consequently, other mechanisms are likely involved - and since Scribble features four PDZ domains with the capacity to interact with interactors, differential regulation of interactions could be based on PDZ-binding groove accessibility. Such a mechanism would provide additional complexity and the ability to tune interactions in addition to competitive binding, which is limited by the fact that the majority of Scribble interactors do not differ significantly enough in their affinities [19–24,26]. Furthermore, there is evidence that Scribble has the capacity to modulate interactions within the context of multiple PDZ domains, such as PDZ3 and PDZ4 which were observed to form a supramodule feature a single active binding site. An interacting peptide primarily bound to the αB/βB pocket of PDZ3, with PDZ4 elongating the binding site within the supramodule [94] instead of providing a discrete second binding site. Other modes of supertertiary structural regulation of interactions have also been observed for other PDZ adaptor proteins [95], and may be applicable for Scribble as well as other polarity signalling proteins.

An additional level of complexity for cell polarity regulation is provided by post-translational modifications such as via phosphorylation of PBMs [24,96,97], as well as the growing number of PBM interactions that do not fit the typical three classes [98–101]. One can envisage an overall system of Scribble regulation features multiple levels; PDZ binding grove accessibility, PBM affinity, post-translation modifications and cellular localisations [40,91,94,96–98].

Viral effector proteins such as HPV E6, HTLV-1 Tax, IAV NS1, and TBEV NS5 target Scribble to influence tissue architecture and integrity, however, there is a growing number of viruses that deploy cell polarity disrupting proteins which have yet to be functionally analysed [34–36,102]. Large-scale phage-display mapping of PDZ domain interactions identified rh11 from the Rhesus Cytomegalovirus, m147R from the Myxoma virus, gp147R from Rabbit fibroma virus, us32 from Cytomegalovirus [103] as potential host cell polarity signalling subverting proteins, and future investigations of their potential interaction partners may shed more light on the precise mechanisms of polarity signalling in healthy and virally infected cells. Future mechanism-based studies of the intricacies of viral subversion of host cell polarity signalling will continue to provide new insights into the workings of cell polarity signalling both in healthy and diseased states and serve as platforms for the development of therapeutic agents that exploit cell polarity signalling for viral infections as well as virus-associated malignancies [4].

## Perspectives

- Viruses subvert Scribble mediated polarity signalling, leading to diverse outcomes including increased virulence as well as increased tumourigenesis in virus-associated malignancies. This underscores the centrality of polarity signalling for tissue and organismal health and well-being
- Scribble is a highly connected polarity adaptor protein involved in numerous signalling pathways and sits at the centre of a substantial interaction network, which is disrupted by viral effector molecules. It is likely that disruption to host cell polarity signalling is not solely based on viral effectors outcompeting endogenous Scribble partners, and instead outcomes such as altered Scribble localisation, post-translational modifications and allosteric control are likely to play an important role.
- The pivotal role of Scribble in polarity signalling raises the possibility of next-generation therapeutics that modulate polarity signalling both in the context of acute viral infections as well as the development of virus-associated malignancies. Whilst significant challenges remain to be overcome for targeting PDZ domains such as those of Scribble for therapy, mastering these challenges may provide substantial clinical benefits.

## Open Access

Open access for this article was enabled by the participation of La Trobe University in an all-inclusive *Read & Publish* agreement with Portland Press and the Biochemical Society under a transformative agreement with CAUL.

## Abbreviations

APC: antigen-presenting cell
EMT: epithelial to mesenchymal transition
HCV: Hepatitis C virus
HPV: Human Papillomavirus
HTLV-1: Human T-lymphotropic virus 1
IAV: Influenza A virus
ITC: isothermal titration calorimetry
LRR: leucine rich repeats
MTase: methyltransferase
NS5: non-structural 5
PBM: PDZ Binding Motif
PDZ: PSD-95/Discs-large/ZO-1
TBEV: Tick-borne encephalitis virus
